# Effects of SRI-32743, a Novel Quinazoline Structure-Based Compound, on HIV-1 Tat and Cocaine Interaction with Norepinephrine Transporter

**DOI:** 10.3390/ijms25147881

**Published:** 2024-07-18

**Authors:** Ana Catya Jiménez-Torres, Katherine D. Porter, Jamison A. Hastie, Charles Adeniran, Omar Moukha-Chafiq, Theresa H. Nguyen, Subramaniam Ananthan, Corinne E. Augelli-Szafran, Chang-Guo Zhan, Jun Zhu

**Affiliations:** 1 Department of Drug Discovery and Biomedical Sciences, College of Pharmacy, University of South Carolina, 715 Sumter Street, Columbia, SC 29208, USA; aj83@mailbox.sc.edu (A.C.J.-T.); kdp3@email.sc.edu (K.D.P.); jhastie@mailbox.sc.edu (J.A.H.); 2 Department of Pharmaceutical Sciences, College of Pharmacy, University of Kentucky, Lexington, KY 40536, USA; charles.adeniran@outlook.com (C.A.); zhan@uky.edu (C.-G.Z.); 3 Molecular Modeling and Biopharmaceutical Center, College of Pharmacy, University of Kentucky, Lexington, KY 40536, USA; 4 Department of Chemistry, Scientific Platforms Division, Southern Research, Birmingham, AL 35205, USA; omoukha-chafiq@southernresearch.org (O.M.-C.); tnguyen@southernresearch.org (T.H.N.); sam.ananthan@nih.gov (S.A.); caugelli-szafran@southernresearch.org (C.E.A.-S.)

**Keywords:** norepinephrine transporter, dopamine, HIV-Tat protein, uptake, cocaine, amphetamine

## Abstract

Prolonged exposure to HIV-1 transactivator of transcription (Tat) protein dysregulates monoamine transmission, a physiological change implicated as a key factor in promoting neurocognitive disorders among people living with HIV. We have demonstrated that in vivo expression of Tat in Tat transgenic mice decreases dopamine uptake through both dopamine transporter (DAT) and norepinephrine transporter (NET) in the prefrontal cortex. Further, our novel allosteric inhibitor of monoamine transporters, SRI-32743, has been shown to attenuate Tat-inhibited dopamine transport through DAT and alleviates Tat-potentiated cognitive impairments. The current study reports the pharmacological profiles of SRI-32743 in basal and Tat-induced inhibition of human NET (hNET) function. SRI-32743 exhibited less affinity for hNET binding than desipramine, a classical NET inhibitor, but displayed similar potency for inhibiting hDAT and hNET activity. SRI-32743 concentration-dependently increased hNET affinity for [^3^H]DA uptake but preserved the V_max_ of dopamine transport. SRI-32743 slowed the cocaine-mediated dissociation of [^3^H]Nisoxetine binding and reduced both [^3^H]DA and [^3^H]MPP+ efflux but did not affect d-amphetamine-mediated [^3^H]DA release through hNET. Finally, we determined that SRI-32743 attenuated a recombinant Tat_1–86_-induced decrease in [^3^H]DA uptake via hNET. Our findings demonstrated that SRI-32743 allosterically disrupts the recombinant Tat_1–86_–hNET interaction, suggesting a potential treatment for HIV-infected individuals with concurrent cocaine abuse.

## 1. Introduction 

The introduction of combined antiretroviral therapy (cART) significantly increases the life expectancy of individuals positive for human immunodeficiency virus (HIV). However, the expectation that reducing the central nervous system (CNS) viral load might lead to a reduction in neurodegeneration and the occurrence of HIV-associated neurocognitive disorders (HAND) is still under investigation [1,2]. The prevalence of HAND is still detectable in people being treated with cART. Cognitive impairment, including asymptomatic or milder forms of HAND, affects nearly 39% of people living with HIV under cART [3], indicating that the viral load is not the only factor underlying the pathophysiology of these neurocognitive disorders. 

Potential factors independent of viral load involved in the development of HAND include the released HIV-viral proteins, neuroinflammatory process, and the imbalance of the dopaminergic neurotransmission [4,5,6,7]. HIV-1 transactivator of transcription (Tat) protein is the first viral protein to be translated after viral integration. It is secreted from HIV-infected cells and reaches the CNS in early infection [8,9,10]. Tat production is not affected by HIV protease inhibitors [9], and Tat remains biologically active, acting as an extracellular toxin [11]. 

The dopaminergic system is involved in learning, memory, reward, motivation, and psychomotor functions. Clinical observations, postmortem examinations, and in vitro studies indicate that dopamine (DA) imbalance occurs at early and advanced stages of HIV infection [12,13,14,15]. At early stages, HIV infection increases extracellular levels of DA [12]. However, low levels of DA [13,14] and dopaminergic neuron damage occur in the advanced stages [16,17].

The homeostasis of DA levels is mainly mediated by the presynaptic membranal DA transporter (DAT), which is critical for DA reuptake. Previously, it has been reported that Tat induces inhibition of DAT [18,19,20] by its binding to allosteric binding sites in the extracellular region of the transmembrane domain of DAT [21]. 

The disruption of DA reuptake [18] and the increase in extracellular DA levels [22], in parallel with a reduction of learning and memory functions, were reported in inducible Tat transgenic (iTat-tg) mice [23]. In the earlier studies, we demonstrated that a novel quinazoline structure-based compound, SRI-32743, normalizes Tat-induced inhibition of DAT and reduces both cocaine reward enhancement and cognitive impairment in inducible Tat transgenic mice [23]. DA clearance in caudate and nucleus accumbens is mediated mainly by DAT; nevertheless, DA reuptake in the frontal cortex, a region with reciprocal connections with limbic formations (hippocampus, hypothalamus, and the amygdala) that plays a key role in cognitive processes and motivation, depends primarily on the norepinephrine transporter (NET) [24]. Our recent studies using molecular dynamic simulations and side-directed mutagenesis have demonstrated that HIV-1 Tat binds to the outward-open state of hNET and the tyrosine467 hNET functions as a key residue that influences Tat-induced inhibition of DA transport [25]. 

This current study was designed to determine whether SRI-32743 allosterically modulates the DA transport via NET, as it does for DAT [23]. Based on the monoamine hypothesis for cognitive impairments, our approach lends further support for pursuing the development of allosteric modulators of DAT and NET as a therapeutic approach for mitigating Tat-induced neurocognitive disorders in the context of drug abuse in HIV^+^ patients.

## 2. Results

### 2.1. Inhibition Effects of SRI-32743 on WT hNET 

**Figure 1:**
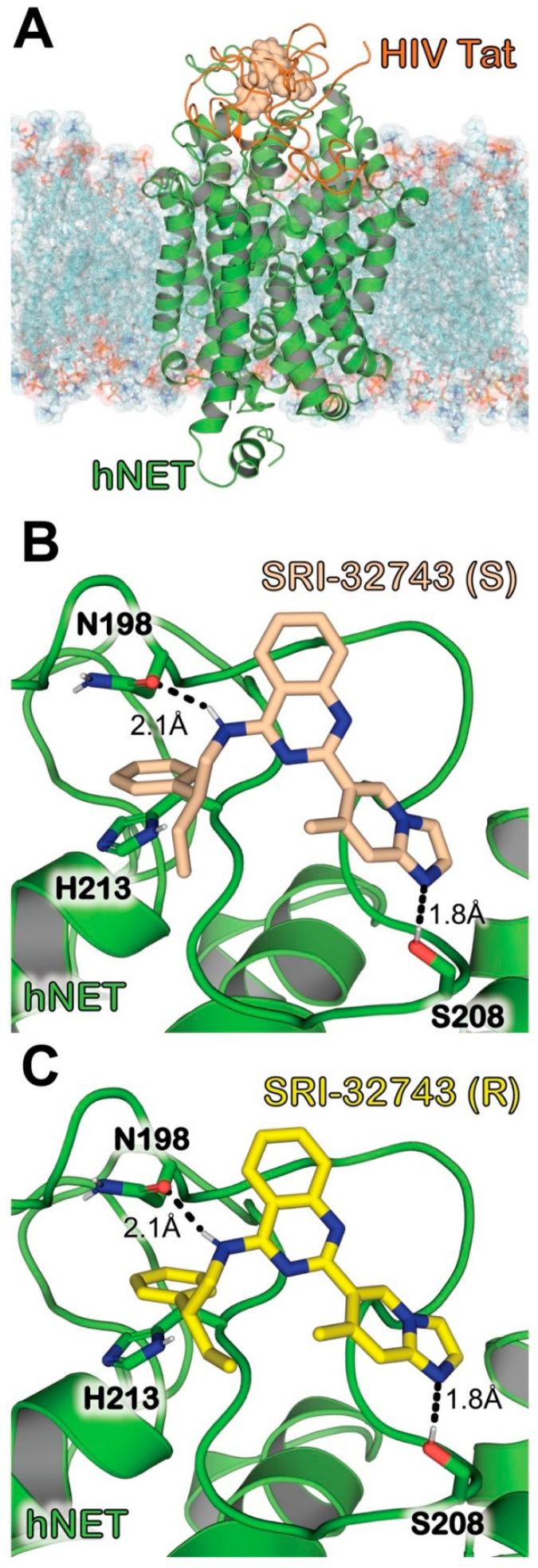
Molecular docking structure of SRI-32743 with hNET. (**A**) Global view showing HIV-1 Tat, hNET, and SRI-32743 in a POPC lipid bilayer. HIV-1 Tat (orange) and hNET (green) are represented in the cartoon model. The lipid bilayer is represented as sticks and spheres, colored according to heavy atoms. Note: HIV-1 Tat was not initially included in the system for molecular docking and energy minimization. (**B**) A closer view of the binding position for (S)-(−)-SRI-32743 bound to hNET at a location that interferes with HIV-1 Tat binding to hNET. (S)-(−)-SRI-32743 (wheat) is represented with the sphere model. hNET (green) is represented in the cartoon model and amino acid residues are represented in the stick model. (**C**) A closer view of the binding pose of (R)-(+)-SRI-32743 bound to hNET at a location that interferes with HIV-1 Tat binding to hNET. (R)-(+)-SRI-32743 (yellow) is represented with the sphere model. Residues N198 and S208 (red) are shown as sticks which are important for binding interaction between hNET (green) and SRI-32743. Intermolecular hydrogen bonding interactions are indicated as dashed lines, and distances are labeled next to the respective lines.

**Figure 2:**
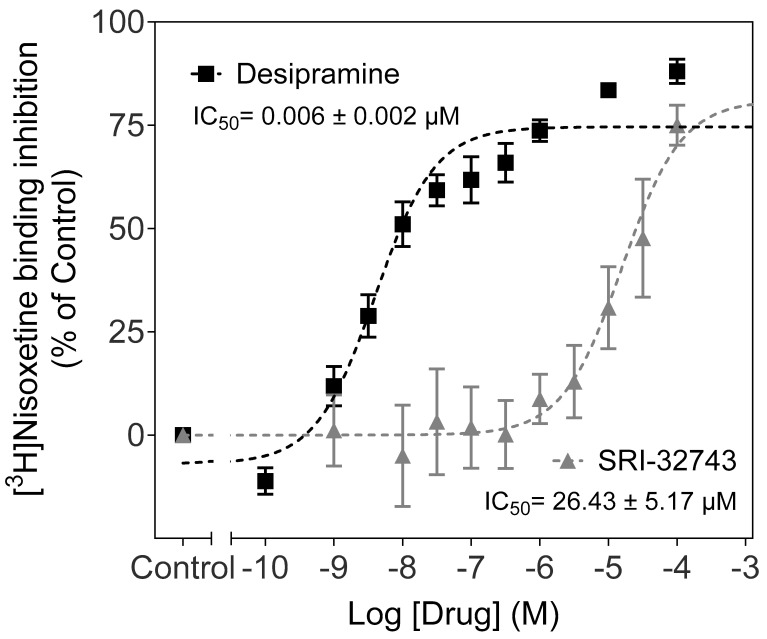
Inhibitory effect of SRI-32743 on [^3^H]Nisoxetine binding in CHO-K1 intact cells expressing WT hNET. Dose–response curves for desipramine or SRI-32743 were fit to a one-component equation conducted by duplication in CHO-K1 intact cells transiently transfected with WT hNET. Specific binding was determined in the presence of [^3^H]Nisoxetine (0.5 nM). For non-specific binding, cells were incubated with 10 µM of desipramine and mazindol (2017 ± 467 DMP). Data are presented as means ± S.E.M. of three or four separate experiments, in duplicate.

Based on the modeled binding complex structure shown in Figure 1, we tested the inhibitory potency of SRI-32743 on [^3^H]DA uptake in CHO-K1 cells expressing wild-type (WT) hDAT or WT hNET. A paired Student’s *t*-test revealed that SRI-32743 displayed an IC_50_ value (8.16 ± 1.16 µM) in inhibiting DA uptake via WT hDAT, similar (*p* = 0.33) to its potency (12.03 ± 3.22 µM) in inhibiting DA uptake via WT hNET. Further, we determined the inhibitory potency of SRI-32743 on [^3^H]Nisoxetine binding in CHO-K1 expressing WT hNET. As reported in Figure 2, SRI-32743 displayed a lower inhibitory potency (IC_50_ value = 26.43 ± 5.17 µM) than desipramine (IC_50_ = 6.0 ± 4.0 nM), a NET inhibitor, in inhibiting [^3^H]Nisoxetine binding. Based on these IC_50_ values, we calculated the E_max_ parameters that refer to the maximum attributable effect of SRI-32743. SRI-32743 showed a similar E_max_ range (61.42–66.05%) for [^3^H]DA uptake via both monoamine transporters, WT hNET or WT hDAT. As illustrated in Figure 2, SRI-32743 displayed an E_max_ of 72.09 ± 9.22% for [^3^H]Nisoxetine binding; meanwhile, the E_max_ value for desipramine, a selective NET inhibitor, was 91.73 ± 18.75%. 

### 2.2. Kinetic Effects of SRI-32743 on [^3^H]DA Uptake in WT hNET

**Figure 3:**
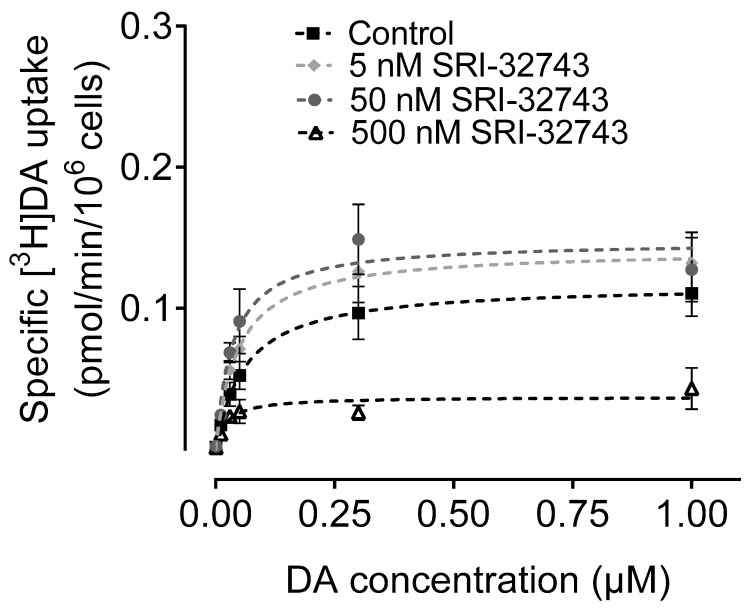
Effects of SRI-32743 on V*_max_* and K*_m_* of [^3^H]DA uptake via hNET in CHO-K1 intact cells. The effect of SRI-32743 at 5, 50, or 500 nM on the specific [^3^H]DA uptake was determined in CHO-K1 cells transiently transfected with WT hNET using six concentrations of DA (0.001–1.0 µM) mixed with a fixed concentration of [^3^H]DA (500,000 dpm/well, specific activity: 21.2 Ci/mmol). In parallel, non-specific uptake of each concentration of [^3^H]DA in the presence of 10 µM of nomifensine and 100 µM desipramine was subtracted from the total uptake for calculating the specific NET-mediated uptake. The curves were calculated by fitting the data to the Michaelis–Menten equation and represent the means ± S.E.M. of four independent experiments, in duplicate.

To determine the effects of SRI-32743 on DA uptake via hNET, we examined the kinetic parameters of [^3^H]DA uptake after treatment with 5, 50, or 500 nM of SRI-32743 in CHO-K1 cells expressing WT hNET. SRI-32743 concentrations were selected based on our previous published data, which showed the allosteric effect of 50 nM SRI-32743 to attenuate the Tat–DAT interaction with any alteration on the basal DA reuptake [23]. As reported in Table 1, the one-way ANOVA revealed significant differences in the V*_max_* (*p* < 0.005) and K*_m_* values (*p* < 0.05) between treatments. Post-hoc Tukey’s multiple comparison analysis showed no changes in the V*_max_* values of [^3^H]DA uptake via hNET after the addition of 5 or 50 nM SRI-32743; however, the *K_m_* values of [^3^H]DA uptake were significantly reduced by 45% at 50 nM. A higher concentration of SRI-32743 (500 nM) significantly decreased both *V_max_* and *K_m_* values, by 66% and 62%, respectively (Figure 3). 

### 2.3. Effects of SRI-32743 on Cocaine-Induced Dissociation of [^3^H]Nisoxetine Binding 

**Figure 4:**
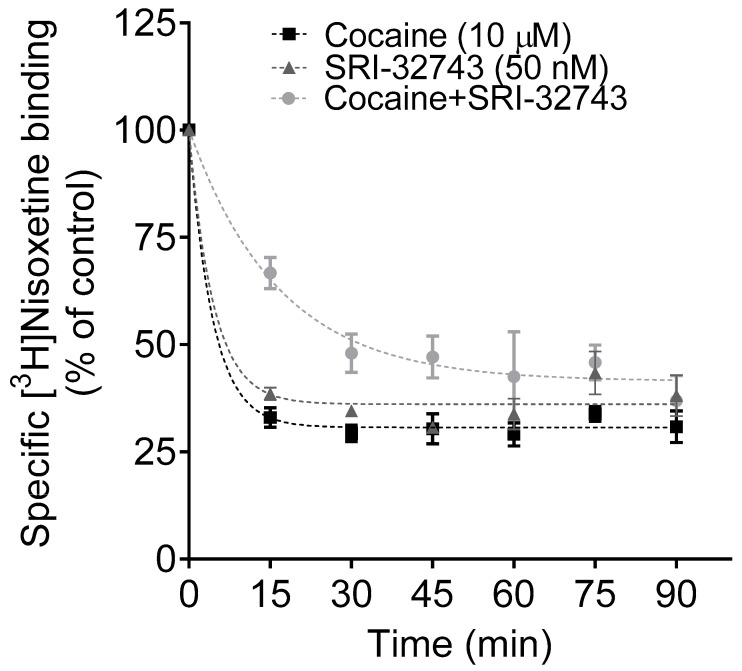
Effect of SRI-32743 on cocaine-mediated [^3^H]Nisoxetine binding dissociation in CHO-K1 cells expressing WT hNET. The effect of 50 nM SRI-32743 on the dissociation rate (K_-1_) of [^3^H]Nisoxetine binding induced by cocaine was determined by the specific [^3^H]Nisoxetine binding (0.5 nM) through nonlinear regression analysis using a single-component dissociation model. For non-specific binding, cells were incubated with 10 µM of desipramine and mazindol (1066 ± 180 DMP). For the data analysis, the 100% control point (no drug) was time point 0 for conditions 1 and 2, and time point 15 min (no drug) for condition 3. Each data point is the mean ± S.E.M. of three separate experiments, in triplicate.

[^3^H]WIN35,428 and [^3^H]Nisoxetine are potent reuptake inhibitors with high affinity for DAT and NET, respectively. To determine whether SRI-32743 allosterically modulates NET function, the dissociation of [^3^H]Nisoxetine was examined in CHO-K1 cells expressing WT hNET in the presence of cocaine, SRI-32743 (50 nM), or cocaine + SRI-32743 (Table S1). As reported in Figure 4, the dissociation of [^3^H]Nisoxetine binding with cocaine was well described by a single-component dissociation model (K_−1_ = 0.233 ± 0.021 min^−1^). The unpaired Student’s *t*-test revealed that the addition of 50 nM SRI-32743 following the addition of cocaine significantly slowed the dissociation rate (K_−1_ = 0.087 ± 0.028 min^−1^; *p* = 0.013) compared to cocaine alone. Moreover, SRI-32743 significantly slowed the dissociation of [^3^H]Nisoxetine by itself compared to cocaine alone (K_−1_ = 0.172 ± 0.004 min^−1^; *p* = 0.043) and it was also described by a single-component dissociation model.

### 2.4. SRI-32743-Mediated [^3^H]DA and [^3^H]MPP^+^ Efflux

**Figure 5:**
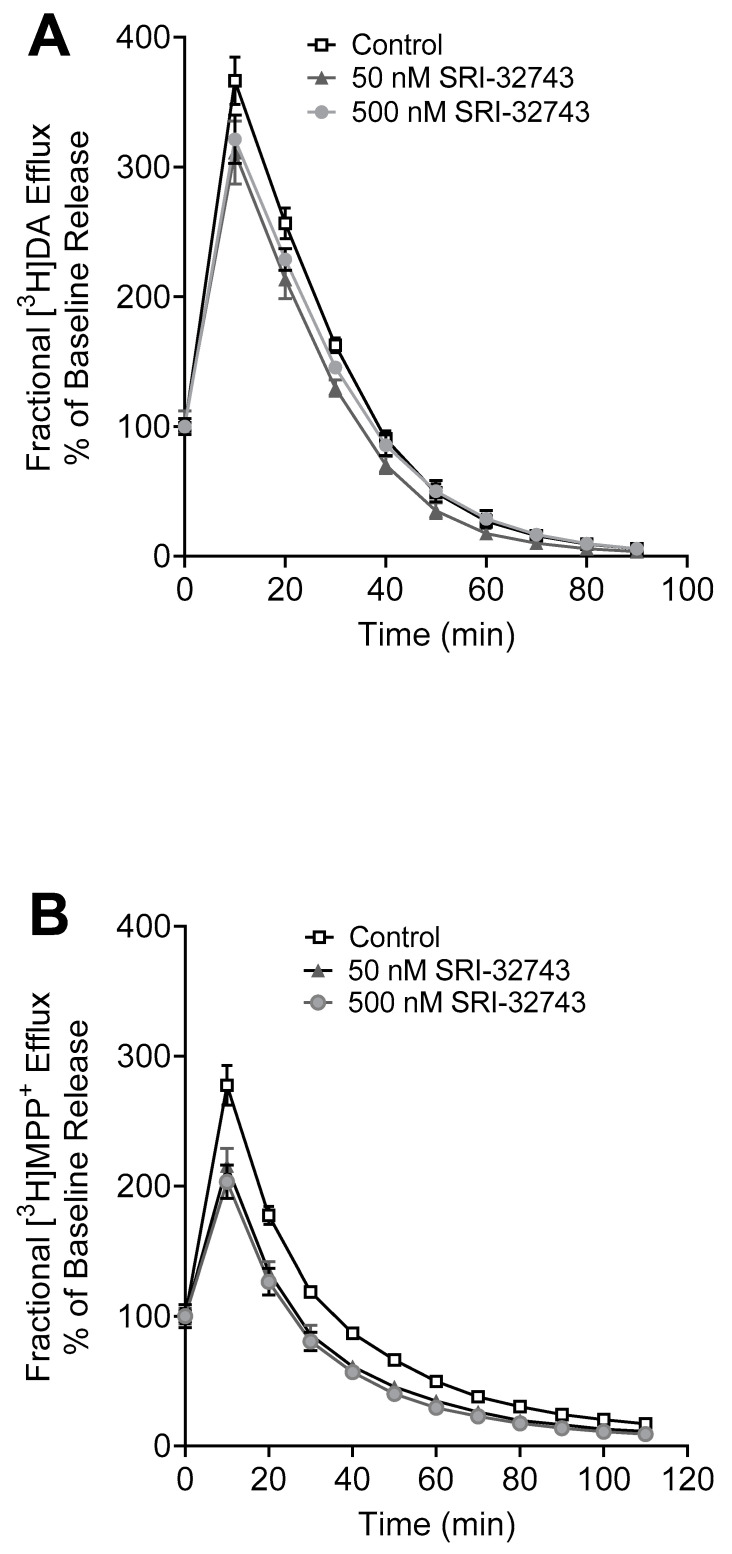
Effects of SRI-32743 on DA and MPP^+^ efflux. CHO cells transfected with WT hNET were pre-incubated with assay buffer in the presence or absence of 500 nM or 50 nM SRI-32743 with 50 nM [^3^H]DA for 20 min or 5 nM [^3^H]MPP^+^ for 30 min at room temperature. After incubation, cells were washed and incubated with fresh buffer. At each indicated time point, the buffer was removed and replaced, and the radioactivity was counted. Fractional efflux of [^3^H]DA or [^3^H]MPP^+^ is expressed as a percentage of the baseline efflux at the zero time point at the beginning of the collection period. (**A**) [^3^H]DA plotted as a percentage of baseline release after the 20 min preloading period. (**B**) [^3^H]MPP^+^ efflux plotted as a percentage of baseline release after the 30 min preloading period.

To determine the effects of SRI-32743 on hNET transporter conformational transitions, we examined the fractional efflux levels of [^3^H]DA and [^3^H]MPP^+^ in WT hNET in the presence or absence of 50 or 500 nM SRI-32743. As shown in Figure 5A, the initial fractional efflux samples were immediately collected at zero time points after the 20 min preloading period, establishing baseline release. For the 50 nM SRI-32743 treatment compared to the control, a two-way ANOVA revealed the significant main effects of time (F_(9, 140)_ = 396.6, *p* < 0.0001), treatment (F_(1, 140)_ = 39.28, *p* < 0.0001), and the interaction between time and treatment (F_(9, 140)_ = 4.481, *p* < 0.0001). Meanwhile, for the 500 nM SRI-32743 treatment compared to the control, a two-way ANOVA revealed the significant main effects of time (F_(9, 140)_ = 422.4, *p* < 0.0001), treatment (F_(1, 140)_ = 10.68, *p* = 0.0014), and the interaction between time and treatment (F_(9, 140)_ = 3.00, *p* = 0.0027). 

For [^3^H]MPP^+^ efflux, as shown in Figure 5B, the initial fractional efflux samples were immediately collected at the zero time point after the 30 min preloading period, establishing baseline release. In regard to the 50 nM SRI-32743 treatment compared to the control, a two-way ANOVA revealed the significant main effects of time (F_(11, 71)_ = 304.8, *p* < 0.0001), treatment (F_(1, 71)_ = 76.95, *p* < 0.0001), and the interaction between time and treatment (F_(11, 71)_ = 5.218, *p* < 0.0001). For the 500 nM SRI-32743 treatment compared to the control, a two-way ANOVA revealed the significant main effects of time (F_(11, 71)_ = 267.9, *p* < 0.0001), treatment (F_(1, 71)_ = 103.8, *p* < 0.0001), and the interaction between time and treatment (F_(11, 71)_ = 6.471, *p* < 0.0001). These results showed that SRI-32743 (50 and 500 nM) decreased both basal [^3^H]DA and [^3^H]MPP^+^ efflux via WT hNET compared with the control. 

**Figure 6:**
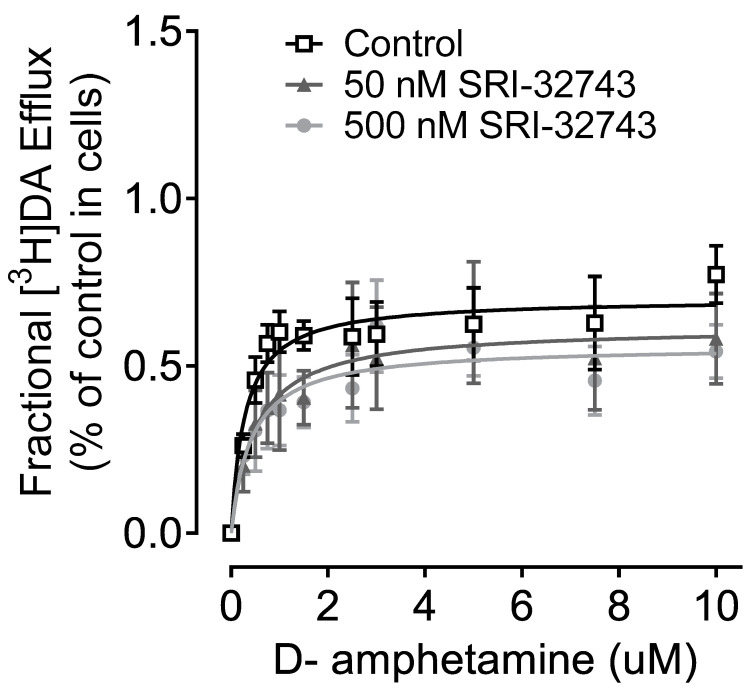
Effect of SRI-32743 on D-amphetamine-induced DA efflux. CHO cells transfected with WT hNET were pre-incubated with assay buffer in the presence or absence of 500 nM or 50 nM SRI-32743 with 50 nM [^3^H]DA for 20 min at RT. After incubation, cells were washed and replaced with fresh buffer every 5 min for 15 min. At 20 min, D-amphetamine (0.25–10 µM) was added, and the same aliquots were taken after 5 min. D-amphetamine-mediated efflux is expressed as means ± S.E.M. of the fractional release of cellular [^3^H]DA per five minutes in the WT hNET control group or SRI-32743 (5 nM or 500 nM).

Dopamine and amphetamine are substrates of hNET [26]. Similar to hDAT, amphetamine induces the reverse transporter, leading to the efflux of DA [26,27]. The effect of SRI-32743 at 50 or 500 nM was tested on D-amphetamine-induced [^3^H]DA efflux via WT hNET. As shown in Table 2 and Figure 6, increasing concentrations of D-amphetamine-induced [^3^H]DA efflux. No significant changes in basal [^3^H]DA efflux were reported in response to SRI-32743 compared with the control group (F_(2, 15)_ = 0.67, *p* = 0.052, one-way ANOVA). 

### 2.5. Effects of SRI-32743 on Tat-Induced Inhibition of [^3^H]DA Uptake via hNET

**Figure 7:**
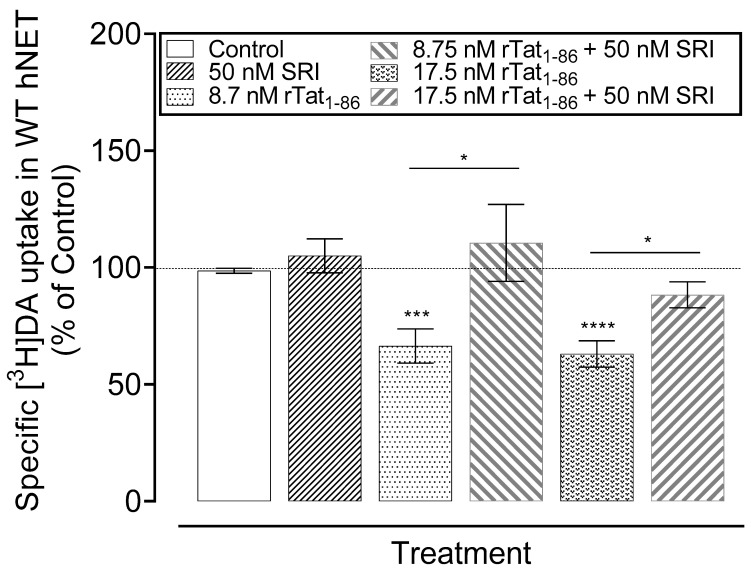
SRI-32743 reduces the effect of Tat-induced inhibition of [^3^H]DA uptake via hNET. CHO-K1 cell suspensions transiently transfected with WT hNET were incubated with or without recombinant Tat_1–86_ (rTat_1–86_, 8.7 or 17.5 nM) or SRI-32743 (50 nM). Specific uptake was determined in the presence of [^3^H]DA (50 nM). For non-specific uptake, cells were incubated with 10 µM of nomifensine and 100 µM of desipramine. Data for _r_Tat_1–86_, SRI-32743, and SRI-32743+ _r_Tat_1–86_ are expressed as the percentage of the respective controls for DA uptake in the absence of _r_Tat_1–86_ and SRI. Data are presented as means ± S.E.M of five separate experiments, in duplicate. Two-way ANOVA followed by Tukey’s multiple comparisons for post hoc analysis. * *p <* 0.05, *** *p <* 0.005, and **** *p <* 0.0005, unpaired Student’s *t*-test compared with the respective controls.

We assessed the effect of SRI-32743 in reducing Tat-induced DA reuptake inhibition via WT hNET in CHO-K1 cells. As reported in Figure 7, the two-way ANOVA revealed a significant main effect of SRI-32743 (F_(1, 24)_ = 13.14, *p* < 0.005). SRI-32743 alone showed no significant differences in the specific [^3^H]DA uptake compared to the control, while recombinant Tat_1–86_ induced a significant inhibitory effect of 33.6% (*t*_(9)_ = 4.79, *p* < 0.01, unpaired Student’s *t*-test) and 36.94% (*t*_(9)_ = 6.83, *p* < 0.001, unpaired Student’s *t*-test) on [^3^H]DA uptake at 8.7 nM and 17.5 nM, respectively, compared to the control. However, a combined exposure of SRI-32743 and Tat (8.7 or 17.5 nM) showed no inhibitory effect on the specific [^3^H]DA uptake compared to the control or compounds alone, indicating that SRI-32743 reduced Tat-induced inhibition of DA reuptake mediated by hNET. 

## 3. Discussion 

This study determined the effect of the novel quinazoline-based compound SRI-32743 on the interaction of HIV-1 Tat and cocaine with WT hNET. Our findings demonstrated that the IC_50_ value for SRI-32743 inhibition of DA uptake via WT hNET was within the range of the IC_50_ value displayed to inhibit WT hDAT activity. SRI-32743 exhibited less affinity for hNET binding compared to desipramine, a classical NET inhibitor. Indeed, our results showed that SRI-32743 concentration-dependently altered the V*_max_* of hNET-mediated DA transport and its affinity. This compound displayed high potency as an allosteric modulator of hNET and the ability to reduce Tat-induced NET inhibition of DA uptake, and to disrupt the cocaine– and Tat–hNET interactions as well. These findings correlate with our published reports, showing dysregulation of DA uptake via both DAT and NET in HIV-1 Tat transgenic mice [18]. Thus, the current study provided new insights for developing atypical modulators to attenuate cocaine- and Tat-mediated inhibition of DA transmission via hDAT and hNET in the context of drug abuse and NeuroHIV.

SRI-32743 interacts with DAT residues that modulate the binding of Tat with DAT and modulates DA uptake in an allosteric manner, leading to a reduction in inhibition of DA uptake by Tat and attenuation of Tat-induced cognitive deficits [23]. Here, we reported the pharmacological profile of SRI-32743 to modulate WT hNET. 

NET mediates NE reuptake into NE neurons; however, it also transports structurally similar substrates, such as DA [28]. hNET shares high homology with hDAT, with almost 89% and 80% identity within the substrate binding sites 1 and 2, respectively [29]. Despite their similar amino acid sequences and predicted topologies, several inhibitors display differences in selectivity for NET over DAT [30,31,32]. Our competitive inhibition assays showed that SRI-32743 displayed similar affinity for inhibiting WT hDAT- and WT hNET-mediated DA uptake and that it partially inhibited DA uptake, with E*_max_* values ranging from 66% inhibition of hDAT to 61% of hNET. However, compared with desipramine, a potent selective inhibitor of NET [32,33], SRI-32743 exhibited a 4000-fold lower affinity to bind to the uptake site in NET. These results suggest that this compound acts as an atypical modulator of NET. 

To gain insights into the mechanism by which SRI-32743 exerts its effects on NET, we evaluated a single concentration of SRI-32743 on a dissociation assay. Cocaine is a competitive inhibitor of outward DA transport at hNET [28]. We demonstrated that 10 µM of cocaine potently increased the dissociation of the selective NE reuptake inhibitor, [^3^H]Nisoxetine. Nevertheless, the addition of SRI-32743 after cocaine slowed the dissociation rate of [^3^H]Nisoxetine binding. Indeed, SRI-32743 (50 and 500 nM) significantly decreased the efflux of [^3^H]DA and [^3^H]MPP^+^ via WT hNET. Both DA and MPP^+^ are substrates of NET, but this transporter exhibits relatively lower V*_max_* for MPP^+^ compared to DAT [34]. These findings support our hypothesis that SRI-32743 allosterically modulates DA transport via hNET. Interestingly, SRI-32743 significantly increased the affinity of hNET for DA uptake in a dose-dependent manner, which correlated with the sigmoidal dose–response curves reported in this study for hNET activity and previously reported for hDAT function and binding, distinct from linear dose–response curves observed for competitive inhibitors, such as cocaine [23,28]. 

Amphetamine interacts with the monoaminergic transporters, the serotonin transporter (SERT), DAT, and NET [27,35]. Amphetamine acts as a substrate to promote the inward-facing conformational changes in the monoamine transporters, inducing monoamine efflux by reversing the direction of transport [27]. Here, our results demonstrated that SRI-32743 (50 and 500 nM) did not alter the potency of d-amphetamine to induce DA efflux, suggesting that SRI-32743 does not alter substrate binding sites in the hNET nor the inward equilibrium of the NET transport. This is in contrast to the effect of cocaine, which blocks the amphetamine-mediated DA release by NET [36]. 

Our previous studies showed that SRI-32743 interacts nearly to the Tat binding site on hDAT [23]. Molecular modeling predicted the Tyr470 residue on hDAT as crucial for HIV-Tat-induced inhibition of DA transport and transporter conformational transitions [21]. Moreover, studies using models of the hDAT-Tat complex structure predicted that the Tyr467 functions as a key residue for HIV-1 Tat’s interaction with hNET and that HIV-1 Tat preferentially binds in the outward-open state [25] and inhibits NET function. In this study, the molecular modeling predicted three additional key residues, Asn198, Ser208, and His213, for HIV-1 Tat’s interaction with hNET (Figure 1). Indeed, in the effort to characterize the molecular mechanism of HIV-1 Tat binding to hNET, our ongoing studies using side-directed mutagenesis have validated the involvement of Asn198 as a functional residue in hNET for Tat-induced inhibition of DA transport. Here, our results showed that low rTat_1–86_ concentrations (8.7 and 17.5 nM) reduced the DA uptake via WT hNET. Similar findings were reported in inducible HIV-1 Tat transgenic mice in ex vivo and in vitro studies. These findings support the notion that a decrease in DA uptake and a concomitant increase in DA levels in the prefrontal cortex results from the interaction of HIV-1 Tat with both DAT and NET [18]. Since dopamine uptake in the frontal cortex depends primarily on hNET [24], it is feasible that this transporter has a notable role in the imbalance of the dopaminergic transmission in HIV-1 infection. 

A relevant finding from the current study is that a nanomolar concentration (50 nM) of SRI-32743 itself did not alter the specific [^3^H]DA uptake; however, SRI-32743 inhibited the Tat-induced reduction in DA uptake in WT hNET. These results are comparable with our recently published work, which demonstrated that the same concentration of this ligand attenuates Tat-induced inhibition of DA uptake by hDAT [23]. The racemic SRI-32743 consists of two enantiomers, (R)-(+)-SRI-32743 and (S)-(−)-SRI-32743. We have identified that both enantiomers display the same binding orientation of the quinazoline template, with a different spatial projection of the substituents at the chiral center. The residues L147, V320, and H318 inside the pocket stabilize the binding through hydrophobic interactions, with the hydrophobic residues of hDAT interacting with the hydrophobic regions of SRI-32743 [23]. In this study, the computational modeling prediction led to a similar finding for the interaction of the ligands at hNET. Both the enantiomers, (R)-(+)-SRI-32743 and (S)-(−)-SRI-32743, are predicted to bind to hNET at key residues, which interferes with HIV-1 Tat binding to hNET. The modeling results indicated that the residues Asn198, His213, and Ser208 are key to stabilizing the interaction of SRI-32743 with hNET. 

Our findings demonstrated that SRI-32743 interacts allosterically with both DAT and NET and disrupts Tat’s interaction with both monoaminergic transporters and attenuates the Tat-mediated inhibitory effect on DA transport. 

## 4. Materials and Methods

### 4.1. Materials 

Chinese hamster ovary cells (CHO-K1, ATCC^®^ CCL-61TM) were obtained from ATCC (Manassas, VA, USA). Dihydroxyphenylethylamine, 3,4-[7-3H] ([^3^H]DA, specific activity 20 Ci/mmol), [N-methyl-^3^H]Nisoxetine hydrochloride ([^3^H]Nisoxetine, specific activity 82.0 Ci/mmol), and N-methyl-4-phenylpyridinium ([^3^H]MPP+, specific activity 82.9 Ci/mmol) were purchased from PerkinElmer Life and Analytical Sciences (Boston, MA, USA). SRI-32743 was synthesized at the Southern Research Institute (SRI; Birmingham, AL, USA). Desipramine hydrochloride, mazindol C-IV, nomifensine maleate salt, cocaine hydrochloride, and other fine chemicals/reagents were from Sigma-Aldrich (St. Louis, MO, USA). Recombinant Tat_1–86_ was purchased from ImmunoDX (Woburn, MA, USA).

### 4.2. Cell Culture and Transfection 

Intact CHO-K1 cells were maintained in F-12K medium supplemented with 10% fetal bovine serum (FBS) and 100 U/mL penicillin and streptomycin. Cells were cultured at a density of 1 × 10^5^ cells/well at 37 °C in 5% CO_2_ and transfected with WT hNET or WT hDAT (0.8 µg/well), subcloned into pcDNA3.1+ using Lipofectamine 2000 (Life Tech, Carlsbad, CA, USA). Cells were used for the experiments after 24 h of transfection. 

### 4.3. Competitive Inhibition [^3^H]DA Uptake Assay

The [^3^H]DA uptake was performed in CHO-K1 cells expressing WT hNET, as reported previously [23]. Cells were pre-incubated with selected concentrations of SRI-32743 in Krebs–Ringer–HEPES (KRH) buffer for 10 min at room temperature (RT) and incubated for 8 min after the addition of 50 nM of [^3^H]DA (specific activity 20 Ci/mmol, PerkinElmer Life and Analytical Sciences, Boston, MA, USA). Non-specific [^3^H]DA uptake was determined in the presence of 100 µM of desipramine and 10 µM of nomifensine.

To determine the effect of SRI-32743 on Tat-induced inhibition of DA uptake via hNET, the assay was performed in CHO-K1 cell suspension expressing WT hNET in the presence of 50 nM of SRI-32743 and 8.7 or 17.5 nM of recombinant Tat_1–86_ at RT for 20 min. The Tat concentration was chosen based on our previous report. Cell suspension was incubated for 8 min after the addition of 50 nM of [^3^H]DA at RT. Non-specific [^3^H]DA uptake was determined in the presence of desipramine (10 µM) and nomifensine (10 µM). Cell suspension was washed with ice-cold KRH buffer containing pyrocatechol (1 mM), followed by filtration through Whatman GF/B glass fiber filters (presoaked with 1 mM of pyrocatechol for 3 h) using a Brandel cell harvester (Model MP-43RS; Biochemical Research and Development Laboratories Inc., Gaithersburg, MD, USA). Radioactivity was determined by liquid scintillation spectrometry (model Tri-Carb 2900TR; PerkinElmer Life and Analytical Sciences, Waltham, MA, USA).

### 4.4. [^3^H]DA Saturation Analysis

Kinetic studies were conducted in intact CHO-K1 cells expressing WT hNET, as reported previously [21]. Cells were pre-incubated with 5, 50, or 500 nM of SRI-32743 in KRH buffer for 10 min at RT, then cells were incubated with one of six concentrations of unlabeled DA (0.001 nM–1 µM) and a fixed concentration of [^3^H]DA (50 nM, specific activity, 20.0 Ci/mmol; PerkinElmer Life and Analytical Sciences, Boston, MA, USA) at RT for 8 min and terminated by washing with cold KRH buffer. Non-specific uptake of each concentration of [^3^H]DA (in the presence of 100 µM of desipramine and 10 µM of nomifensine) was subtracted from the total uptake to calculate the NET-mediated uptake. Cells were lysed in 500 µL of 1% SDS for 1 h and the radioactivity was measured using a liquid scintillation counter. 

### 4.5. [^3^H]Nisoxetine Binding Assay

Intact CHO-K1 cells expressing WT hNET were washed with binding-assay buffer [18]. Cells were incubated with one of ten concentrations of desipramine or SRI-32743 (0.1 µM–10 µM) in the presence of 0.5 nM of [^3^H]Nisoxetine (specific activity 82.0 Ci/mmol, PerkinElmer Life and Analytical Sciences, Boston, MA, USA) at RT for 15 min. Then, cells were washed with ice-cold assay buffer, lysed by 1% SDS for 1 h, and subjected to liquid scintillation spectrometry measurement. 

For the binding dissociation assay, conditions were selected based on our pilot study, available in the Supplementary Materials. 

### 4.6. [^3^H]DA and [^3^H]MPP^+^ Efflux Assay

[^3^H]DA and [^3^H]MPP^+^ efflux were performed as reported previously [21,37] in CHO-K1 cells transfected WT hNET by preloading with SRI-32743 (50 or 500 nM) and [^3^H]DA (50 nM) for 20 min or [^3^H]MPP^+^ (5 nM, specific activity 82.9 Ci/mmol, PerkinElmer Life and Analytical Sciences, Boston, MA, USA) for 30 min at RT, followed by replacing with fresh buffer at indicated time points. Finally, the buffer in each well was separated from cells, and radioactivity in the buffer and remaining in the cells was counted. Each fractional efflux value of [^3^H]DA or [^3^H]MPP^+^ in WT hNET was expressed as a percentage of total [^3^H]DA or [^3^H]MPP^+^ in the cells at the start of the experiment. Fractional efflux values of [^3^H]DA and [^3^H]MPP^+^ at 1 to 90 min or 1 to 110 min are expressed as the percentage of total [^3^H]DA or [^3^H]MPP^+^ with preloading with 50 or 5 nM, respectively, present in the cells at the start of the experiment. 

To test the effect of SRI-32743 on D-amphetamine-induced DA efflux in CHO-K1 cells expressing WT hNET, similar conditions as previous reports were used [37,38]. After the 20 min time point, cells were incubated with SRI-32743 (50 or 500 nM) or KRH buffer alone and one of ten concentrations of D-amphetamine (0.25–10 nM, Sigma-Aldrich, St. Louis, MO, USA) or control (no D-amphetamine) in each well in duplicate during 5 min. At the end of the collection period, the cells were lysed with 1% SDS, and samples were collected as leftover [^3^H]DA or [^3^H]MPP^+^ remaining in the cells and as D-amphetamine-stimulated DA efflux. 

### 4.7. Data Analysis

Data are presented as mean ± standard error of the mean. GraphPad Prism version 9.1 was used to calculate IC_50_ values for substrates and inhibitors, inhibiting [^3^H]DA uptake or [^3^H]Nisoxetine binding, which were determined from an inhibitory curve using a one-site model with variable slope, using a best-fit nonlinear regression analysis. To analyze the kinetic parameters (V*_max_* and K*_m_*) of [^3^H]DA uptake, best-fit nonlinear regression analysis using a single-site model was conducted for each individual experiment using GraphPad Prism version 9.1. For data involving comparisons between unpaired samples, the unpaired Student’s *t*-test was used to assess differences in pharmacological parameters (K_−1_, K*_m_*, and V*_max_*) between control and drug-treated groups. Log-transformed values of IC_50_ were used for the statistical comparisons. A two-way ANOVA and Tukey’s multiple comparisons post hoc test were used to assess main and significant effects of SRI-32743 to attenuate Tat-induced inhibition of DA uptake; subsequently, we analyzed significant differences between each drug group and control group with the unpaired Student’s *t*-test. 

## 5. Conclusions

The findings from this report, studying the interaction of SRI-32743 with NET, allowed us to identify targets in the brain to attenuate HIV-1 Tat-inhibited NET function. Based on the monoaminergic hypothesis for cognitive impairments, reducing Tat-induced dysregulation of not only DAT but also NET by allosteric modulators established their potential for therapeutic application in HAND. Here, we provided a key chemical structure for developing allosteric modulators with minimal effects on physiological monoamine transmission but effects to diminish Tat- and cocaine-binding to NET. The results of this study raise the potential for developing allosteric modulators of DAT and NET as a novel treatment for mitigating HAND in HIV+ patients with cocaine use comorbidity.

## Figures and Tables

**Table 1:** Effects of SRI-32743 on V*_max_* and K*_m_* of [^3^H]DA uptake via hNET in CHO-K1 intact cells.

SRI-32743 (nM)	V*_max_*	K*_m_*
0 (Control)	0.145 ± 0.010	0.064 ± 0.007
5	0.176 ± 0.011	0.047 ± 0.004
50	0.188 ± 0.031	0.035 ± 0.003 *
500	0.049 ± 0.009 *	0.024 ± 0.009 **

The V*_max_* and K*_m_* values were calculated by fitting the data to the Michaelis–Menten equation and represent the means ± S.E.M. of four independent experiments, in duplicate. * *p* < 0.05 and ** *p* < 0.01 (ordinary one-way ANOVA and post hoc Tukey’s multiple comparison test) compared to the control.

**Table 2:** Effect of SRI-32743 on V*_max_* and K*_m_* of D-amphetamine-induced DA efflux.

SRI-32743 (nM)	V*_max_*	K*_m_*
0 (Control)	0.700 ± 0.045	0.284 ± 0.101
5	0.616 ± 0.082	0.492 ± 0.289
50	0.559 ± 0.057	0.414 ± 0.200

The V*_max_* and K*_m_* values were calculated by fitting the data to the Michaelis–Menten equation. D-amphetamine-mediated efflux is expressed as means ± S.E.M. of the fractional release of cellular [^3^H]DA per five minutes in the WT hNET control group or SRI-32743 (5 nM or 500 nM).

## Data Availability

The original contributions presented in the study are included in the article/Supplementary Materials, further inquiries can be directed to the corresponding author.

## Supplementary Materials

The following supporting information can be downloaded at: https://www.mdpi.com/article/10.3390/ijms25147881/s1.

## List of Abbreviations

cART	Combined antiretroviral therapy
CHO-K1	Chinese hamster ovary cells
CNS	Central nervous system
DA	Dopamine
DAT	Dopamine transporter
HAND	HIV-associated neurocognitive disorders
HIV-1	Human immunodeficiency virus 1
hDAT	Cloned human dopamine transporter
hNET	Cloned human norepinephrine transporter
NET	Norepinephrine transporter
SRI	Southern Research Institute
rTat_1–86_	Recombinant HIV-1 Tat_1–86_
RT	Room temperature
Tat	Transactivator of transcription
WT	Wild type
[^3^H]DA	Dihydroxyphenylethylamine, 3,4-[7-3H]
[^3^H]MPP^+^	N-methyl-4-phenylpyridinium
[^3^H]Nisoxetine	[N-methyl-3H]Nisoxetine hydrochloride
