# Supplementary material for: Effects of SRI-32743, a Novel Quinazoline Structure-Based Compound, on HIV-1 Tat and Cocaine Interaction with Norepinephrine Transporter
Source: Int J Mol Sci. 2024 Jul 18;25(14):7881. doi: 10.3390/ijms25147881 (PMC11277056; doi:10.3390/ijms25147881)
Supplement: Supplementary file 1 [file ijms-25-07881-s001.zip › ijms-3117582-supplementary.pdf]

Effects of SRI-32743, a novel quinazoline structure-based compound, on HIV-1 Tat and cocaine interaction with norepinephrine transporter

Supplemental Material

Cocaine-induced dissociation of [^{3}H]Nisoxetine binding assay in WT hNET

We designed a pilot study to measure the effect of a fixed concentration of cocaine (10 μM) on the dissociation of a range of concentrations of [^{3}H]Nisoxetine (final concentrations 0.5, 1 or 3 nM) in cells expressing WT hNET. Intact cells were incubated with a fixed concentration of [^{3}H]Nisoxetine in assay buffer (final concentration 15 mM Na_2HPO_4, 30 mM NaH_2PO_4, 122 mM NaCl, 5 mM KCl, 1 mM $MgSO_4$, 10 mM glucose, 1 mM CaCl, and10 nM EDTA) in three different conditions: (A) 3 nM at 4 °C for 90 min; (B) 1 nM at 4 °C for 90 min; (C) 0.5 nM at 4 °C for 90 min or (D) 0.5 nM at RT for 90 min. Then, cells were washed twice with ice-cold assay buffer. Nonspecific [^{3}H]Nisoxetine binding was determined by the addition of desipramine and mazindol (10 μM). After that, the experiment was performed as we reported previously [23]. In condition 1 (cocaine only), at the zero-time point, the binding dissociation was initiated by the application of a single concentration of cocaine (10 μM, Sigma-Aldrich, St. Louis, MO, USA). In condition 3 (cocaine + SRI-32743), 10 min after the addition of cocaine, SRI-32743 (50 nM) was then added to minimize any [^{3}H]Nisoxetine re-association. Fifteen, 30, 45, 60, 75, and 90 min later, cells were washed twice with assay buffer, lysed, and counted by liquid scintillation spectrometry. For data analyses, 0 min (no drugs treatment) and 10 min after the application of cocaine were set as 100% to normalize samples at each time point in conditions 1 and 2, respectively. Ordinary one-way ANOVA analysis showed no differences between different concentrations of [^{3}H]Nisoxetine (0.5 nM – 3 nM) after incubation for two hours at 4°C.

However, a paired Student's *t*-test revealed that cocaine significantly increases the dissociation ratio after the cells were preloaded with 0.5 nM [^{3}H]Nisoxetine during 30 min at RT compared to the incubation at 4°C ($p < 0.05$) (**Table S1**). In the present study, we selected condition D to assess the effect of SRI-32743 on cocaine-mediated dissociation of [^{3}H]Nisoxetine binding in WT hNET.

Table S1. Cocaine-mediated [^{3}H]Nisoxetine binding in CHO-K1 cells expressing WT hNET

Condition	Dissociation rate [K_{-1}; min^{-1}]
A<br>3 nM [^{3}H]Nisoxetine during 2h at 4°C,<br>following 10 µM cocaine 90 min at 4°C	0.036 ± 0.013
B<br>1 nM [^{3}H]Nisoxetine during 2h at 4°C,<br>following 10 µM cocaine 90 min at 4°C	0.065 ± 0.006
C<br>0.5 nM [^{3}H]Nisoxetine during 2h at 4°C,<br>following 10 µM cocaine 90 min at 4°C	0.050 ± 0.013
D<br>0.5 nM [^{3}H]Nisoxetine during 30 min at RT<br>following 10 µM cocaine 90 min at RT	0.234 ± 0.020*

Data are presented as means ± S.E.M of three separate experiments by duplicated. *p<0.05 (Student's *t*-test) compared to Condition C.
